# Supplementary material for: Calcium wave dynamics in the embryonic mouse gut mesenchyme: impact on smooth muscle differentiation
Source: Commun Biol. 2024 Oct 7;7:1277. doi: 10.1038/s42003-024-06976-y (PMC11458798; doi:10.1038/s42003-024-06976-y)
Supplement: Supplementary file 2 — Supplementary Material [file 42003_2024_6976_MOESM2_ESM.pdf]

Supplementary Information to: "Calcium wave dynamics in the embryonic mouse gut mesenchyme: impact on smooth muscle differentiation"

Nicolas R. Chevalier[1*], Léna Zig[1], Anthony Gomis[1], Richard J. Amedzrovi[1], Amira El Merhie[1], Laetitia Pontoizeau[2], Isabelle Le Parco[3], Nathalie Rouach[4], Isabelle Arnoux[4], Pascal de Santa Barbara[5], Sandrine Faure[5]

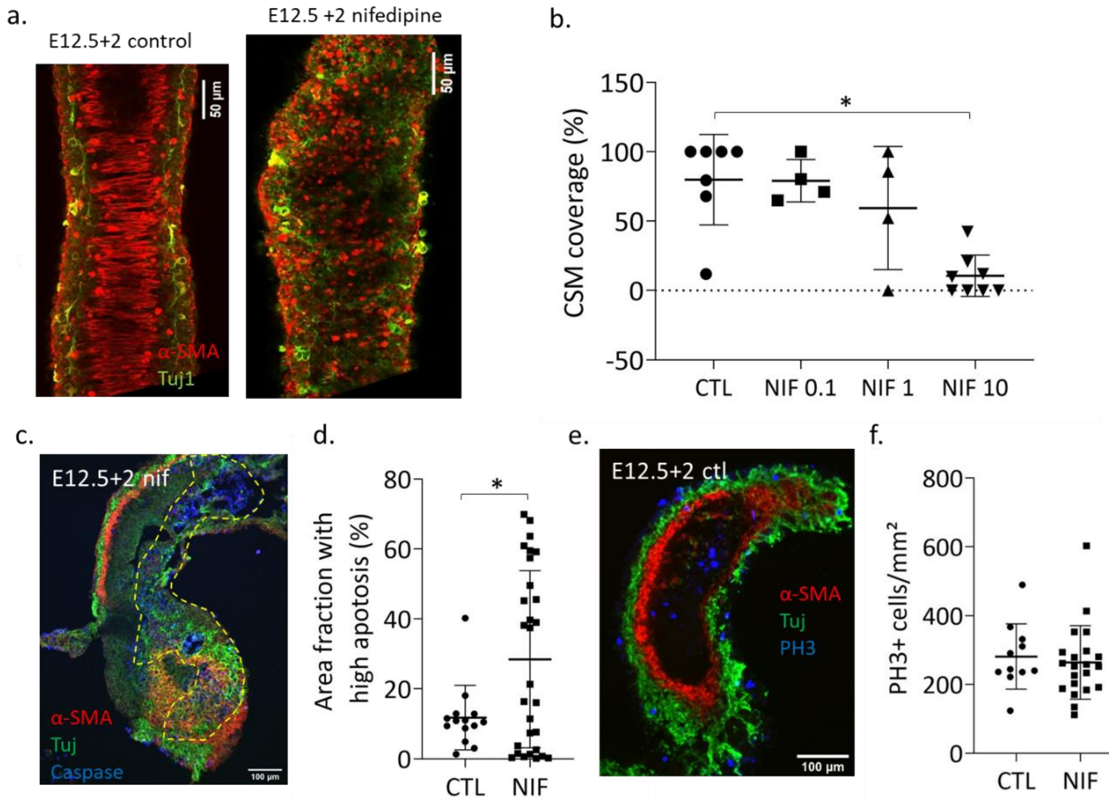

*Figure S1. Effect of nifedipine treatment on E12.5 guts cultured for 2 days. (a) Whole-mount IHC for α-SMA and Tuj1 of control and nifedipine-treated jejunum. Both the ENS and CSM layer are strongly affected by nifedipine treatment. (b) Percent-length of the midgut in which circularly arranged smooth muscle fibers are present at E12.5+2 for control and different concentrations of nifedipine (n=7 DMSO controls, n=4 0.1 µM, n=4 1 µM, n=8 10 µM). (c) α-SMA, Tuj1 and cleaved-caspase-3 frozen section IHC on E12.5+2 gut treated with nifedipine. The dashed yellow region shows an example area with high apoptosis. (d.) Nifedipine treated samples exhibited significantly more tissue fraction area with high apoptosis than controls (controls: n=14 slices analyzed from n=3 guts, nifedipine: n=29 slides from n=4 guts). (e) Example α-SMA, Tuj1 and anti-histone H3 (phospho S10) frozen section IHC on E12.5+2 control gut. (f) The surface density of phospho-histone 3 positive cells was not significantly different between control and nifedipine-treated E12.5+2 samples (controls: n=11 slices analyzed from n=3 guts, nifedipine: n=21 slides from n=3 guts). * p<0.05, Mann-Whitney two-tailed test.*
