# Supplementary material for: Calcium wave dynamics in the embryonic mouse gut mesenchyme: impact on smooth muscle differentiation
Source: Commun Biol. 2024 Oct 7;7:1277. doi: 10.1038/s42003-024-06976-y (PMC11458798; doi:10.1038/s42003-024-06976-y)
Supplement: Supplementary file 3 — Description of Additional Supplementary Materials [file 42003_2024_6976_MOESM3_ESM.pdf]

Description of Additional Supplementary Files

File name: Supplementary Data 1

Description: source data underlying the graphs and charts

File name: Video S1

Description: Evolution of calcium wave activity in the midgut at stages E11.5, E12.5, E14.5 & E14.5+2. All videos are at the same scale and frame rate for comparison.

File name: Video S2

Description: Evolution of calcium wave activity in the colon at stages E12.5, E14.5 & E14.5+2. All videos are at the same scale and frame rate for comparison.

File name: Video S3

Description: Effect of nifedipine 10 µM on calcium waves in E12.5 midgut

File name: Video S4

Description: Effect of BayK 8644 2 µM on calcium waves in E12.5 midgut.

File name: Video S5

Description: Effect of enxolone 33 µM on calcium waves in E12.5 midgut.

File name: Video S6

Description: Effect of ML-7 10 µM on calcium waves in E12.5 midgut.

File name: Video S7

Description: Effect of Y-27632 on contractile waves in a control E12.5+2, a 10 µM E12.5+2 sample, and after increase to 50 µM.

File name: Video S8

Description: Ca2+ activity in a neural crest specific GCaMP sample in E12.5 midgut.
